# Supplementary material for: Sorting at embryonic boundaries requires high heterotypic interfacial tension
Source: Nat Commun. 2017 Jul 31;8:157. doi: 10.1038/s41467-017-00146-x (PMC5537356; doi:10.1038/s41467-017-00146-x)
Supplement: Supplementary file 2 — Supplementary Software 1 [file 41467_2017_146_MOESM2_ESM.zip › PottsModel/SrcPottsModel/doc/index-files/index-16.html]

P-Index


JavaScript is disabled on your browser.


Skip navigation links


- Overview
- Package
- Class
- Use
- Tree
- Deprecated
- Index
- Help

- Prev Letter
- Next Letter

- Frames
- No Frames

- All Classes

A B C D E F G H I J K L M N O P R S T U V W 


## P

paintComponent(Graphics) - Method in class gui.PottsCanvas


Pair<A,B> - Class in model


Pair(A, B) - Constructor for class model.Pair


ParameterManager(Properties) - Constructor for class model.Constants.ParameterManager


parametersToString() - Method in class model.Pixel


params - Variable in class model.Constants


parseArguments(String[]) - Static method in class engine.CommandLineSimulation


perimeterElasticity - Variable in class model.Constants.ParameterManager


PerimeterEnergyFunction - Class in model


PerimeterEnergyFunction() - Constructor for class model.PerimeterEnergyFunction


PerimeterStatistic - Class in engine


PerimeterStatistic(PottsEngine) - Constructor for class engine.PerimeterStatistic


PerimeterStatistic(PottsEngine, int) - Constructor for class engine.PerimeterStatistic


perimeterStd - Variable in class model.Constants.ParameterManager


Pixel - Class in model


Pixel(int, int, Cell, Lattice) - Constructor for class model.Pixel


Pixel(Pixel, Cell) - Constructor for class model.Pixel


Pixel(Pixel) - Constructor for class model.Pixel


Pixel.PixelType - Enum in model


PixelDisplay - Class in gui
:   Abstract class managing active edges and pixel colors.

PixelDisplay(int, int, int) - Constructor for class gui.PixelDisplay
:   Initialize Pixel at location (x, y)

PixelDisplay(int, int, int, Color) - Constructor for class gui.PixelDisplay


PixelInteractionEnergyFunction - Class in model


PixelInteractionEnergyFunction() - Constructor for class model.PixelInteractionEnergyFunction


PixelIterator() - Constructor for class model.Lattice.PixelIterator


PixelShape - Interface in gui
:   Graphics Managment.

PixelShape.Edge - Enum in gui


PixelShape.Type - Enum in gui


pixelType - Variable in class model.Constants.ParameterManager


PlotPanel - Class in gui


PlotPanel(List<Statistic>) - Constructor for class gui.PlotPanel
:   Constructs a new demonstration application.

pointTowards(Point) - Method in class model.FiniteVector
:   Length is unchanged

pointTowards(Point) - Method in class model.HalfLine
:   Length is unchanged

pointTowards(Point) - Method in interface model.Vector


PottsCanvas - Class in gui
:   Graphical representation of the Potts Model Lattice.

PottsCanvas(int, int, Constants) - Constructor for class gui.PottsCanvas
:   Constructs a Cartesian grid that can carry (pPottsWidth x pPottsHeight)
    PottsPixels a.k.a Spins.

PottsEngine - Class in engine
:   Class describing core loop functionality of the PottsModel simulation.

PottsEngine(Constants[]) - Constructor for class engine.PottsEngine


PottsEngine(Constants) - Constructor for class engine.PottsEngine


PottsEngine(Simulation) - Constructor for class engine.PottsEngine


PottsEngine(Constants, Lattice) - Constructor for class engine.PottsEngine


PottsEngine.State - Enum in engine
:   Enum class representing the different states of the potts engine that can
    be passed as a notification message to the different class observers.

PottsEngine.StateVariables - Class in engine
:   Initializes state variables (if they are objects, they are set to null)

PottsFrame - Class in gui


PottsFrame(PottsEngine) - Constructor for class gui.PottsFrame


PottsFrame.Action - Enum in gui


PottsLogger - Class in engine
:   Potts Automaton Logger.

PottsLogger() - Constructor for class engine.PottsLogger


PottsToolbar - Class in gui


PottsToolbar(PottsFrame) - Constructor for class gui.PottsToolbar


propertyChange(PropertyChangeEvent) - Method in class gui.PottsCanvas

A B C D E F G H I J K L M N O P R S T U V W

Skip navigation links


- Overview
- Package
- Class
- Use
- Tree
- Deprecated
- Index
- Help

- Prev Letter
- Next Letter

- Frames
- No Frames

- All Classes
